# Evidence of User-Expert Gaps in Health App Ratings and Implications for Practice

**DOI:** 10.3389/fdgth.2022.765993

**Published:** 2022-02-17

**Authors:** Pier-Luc de Chantal, Alexandre Chagnon, Michael Cardinal, Julie Faieta, Alexandre Guertin

**Affiliations:** ^1^Department of Psychology, Université du Québec à Montréal, Montréal, QC, Canada; ^2^Therappx, Granby, QC, Canada; ^3^Department of Pharmacy, Université Laval, Québec City, QC, Canada; ^4^Department of Rehabilitation Science and Technology, University of Pittsburgh, Pittsburg, PA, United States

**Keywords:** digital health, mobile applications, mental health, physical health, delivery of health care, user ratings, expert ratings, app stores

## Abstract

Searching the commercial Google Play Store and App Store is one of the most common strategies for discovering mobile applications for digital health, both among consumers and healthcare professionals. However, several studies have suggested a possible mismatch between this strategy and the objective of finding apps in physical and mental health that are both clinically relevant and reliable from a privacy standpoint. This study provides direct evidence of a gap between the five-star user rating system and expert ratings from a curated library of over 1,200 apps that cover both physical and mental health. An objective metric is derived to assess the strength of the user-expert gap for each app, which in turn allows identifying missed opportunities—low user ratings and high expert ratings—and overrated apps—high user ratings and low expert ratings. Implications for practice and care delivery are discussed.

## Introduction

The emergence of technology has reinvented the way consumers and professionals view the delivery of physical and mental health services. Both have welcomed the potential of mobile applications to enhance health services and to overcome key barriers to accessing care (1). Yet, intentions and actions are often disconnected. Despite growing consumer interest, the vast majority find health apps on social media or through personal searches in commercial app stores, with a small minority of them being recommended by a healthcare professional (2). One of the most cited barriers to adoption by healthcare professionals is the difficulty of finding reliable information on efficacy, privacy, and security (3).

In the absence of easy access to reliable information, searching the commercial app stores remains a common method to discover and select health apps, even among experienced professionals (3). This strategy can yield search results very rapidly, but professionals are likely to encounter misinformation and make decisions based on misleading clues. In a recent review of top-ranked mobile applications for mental health, researchers found that a third of apps whose descriptions mentioned “scientific techniques” referred to principles that are not truly evidence-based (4). Even more problematic is the reliance on user ratings as a reliable estimate for quality assessment. In fact, there are reports that some developers engage in dishonest behavior aimed at manipulating user reviews and five-star ratings (5). Despite being clinically problematic or even potentially harmful, several health apps enjoy very favorable user reviews and ratings. One striking example is *Instant Blood Pressure*, a highly rated app which was taken off the market shortly after its inaccuracy was spotted by researchers (6, 7). Yet the five-star value of a health app remains one of the best predictors of consumer adoption and the most important driver of downloads (8).

When selecting health apps, healthcare professionals share that they are looking for safe, private, clinically relevant, and evidence-based apps (9). However, such information is relatively scarce and difficult to identify in commercial app stores. New curated libraries have been developed to help professionals navigate the complex and rapidly changing digital health ecosystem. Platforms such as the APA library^1^ (10), ORCHA library,^2^ PsyberGuide,^3^ MindTools^4^ and Therappx's Core library^5^ offer objective evaluation frameworks that provide “expert” guidance and information on health apps usability, clinical value, supporting evidence and data rights. The present study compares information in Therappx's Core library (Core) with that in app stores. Understanding the relationship between expert ratings found on curated libraries free from commercial bias with developers, and user ratings found on commercial stores helps to isolate the differential value of each in informing professional choice.

Prior investigations have already suggested gaps between expert and user ratings. For instance, having a healthcare organization involved in the development of the app, which is generally rated favorably by professionals, is not associated with positive ratings nor downloads from consumers when it comes to maternal and infant apps (11). There is also partial evidence of a weak correlation between user ratings and clinical utility rated by experts (1), suggesting that the two reflect competing priorities [for similar results regarding apps for women's health, see (9)]. Only Lagan et al. explicitly compared five-star user ratings provided in commercial stores with expert ratings from a curated library of 278 apps for mental health (12). They confirmed user-expert gaps for key clinical metrics such as privacy, security, efficacy, and engagement. However, no study has yet quantified the strength of user-expert gaps in a way that provides actionable insights into health app selection and the prescribing process, nor has any study provided information on user-expert gaps outside the domain of mental health. The first objective of this paper is therefore to quantify the overlapping characteristics of expert and user ratings for both physical and mental health apps. The second objective to provide a key metric for identifying missed opportunities—low user ratings and high expert ratings—and overrated apps—high user ratings and low expert ratings.

## Materials and Methods

### Therappx's Core Library

Core is a curated library developed by Therappx, a Canadian company specializing in expert assessment of health apps. Core's framework provides expert reviews and information on data privacy, usability, clinical qualities and supporting evidence from clinical trials or feasibility studies. It should be noted, however, that the quality of the evidence is not assessed during the review process. The standardized assessment follows existing recommendations by incorporating elements derived from validated rating scales, such as the Mobile Application Rating Scale (13) and the App Behavior Change Scale (ABACUS) (14). Each of the 1,761 health apps found in the library is reviewed by a trained librarian and a clinical professional, together generating more than 100 data points per app. Previous unpublished data^6^ showed good interrater reliability for key clinical variables such as the ABACUS and the care continuum component of the assessment, with Cronbach's alpha of 0.75 and 0.90, respectively. Core is designed to be used by healthcare professionals and provides raw data points in a user-friendly interface. It also facilitates searching for the most relevant app for any given clinical situation by providing professionals with advanced search functionalities. Core doesn't provide an explicit quantitative expert score. Instead, health apps are assigned a relative ranking position which is derived from the data rights, usability, clinical and evidence subscales. Because the subscales are calculated from a varying number of data points, and therefore their absolute range differs, each is standardized and summed to produce an overall expert ranking. This ranking system was designed to apply filters and identify the most adequate health apps relative to others in the database.

### Health Apps Dataset

A total of 1,233 health apps for which both the five-star user rating and expert rating were available were extracted from the Core database on June 3th, 2021. The dataset included the health domain covered (physical health, mental health), operating system (Android, iOS), five-star user rating, number of reviews, expert rating and scores of the data rights, usability, clinical and evidence subscales. Table 1 shows descriptive statistics for user and expert ratings. Of the 1,233 apps, 687 targeted a physical health condition and 546 a mental health condition, and a total of 614 were Android apps and 619 were iOS apps. It should be noted that Core includes overlaps of the same health apps between stores, and reconciliation is not feasible due to the architecture of the database. Therefore, discrepancies between Android and iOS versions of the same app are not explicitly analyzed in this study.

**Table 1:** Descriptive statistics for user and expert ratings (*n* = 1,233).

	**M**	**SD**	**Min**	**Max**	**Skewness**	**Kurtosis**
User rating	4.23	0.70	1.00	5.00	−1.70	3.57
Expert rating	0.00	2.59	−11.18	9.39	0.27	0.58
Data rights	3.08	2.84	−9	10	−1.30	3.07
Usability	3.59	2.42	−6	9	0.13	0.13
Clinical	2.21	2.49	−5	7	0.32	−0.67
Evidence	0.34	0.72	0	4	2.32	5.23

*The expert rating is the sum of the standardized subscales. M, Mean. SD, Standard deviation*.

### Data Analysis

The existence of user-expert gaps in health apps ratings was assessed using a Bayesian inference approach. This makes it possible to compare the marginal likelihoods between a null and an alternative hypothesis. Here, the null hypothesis (H0) would be that there is no relationship between user and expert ratings, i.e., a user-expert gap. The alternative hypothesis (H1) would be that there is a relationship between the two rating systems, thus providing evidence against a user-expert gap. We performed linear regressions to gain a more nuanced understanding of the relationship between each expert rating subscale and user rating.

To identify missed opportunities (i.e., positive expert ratings and negative user ratings) and overrated apps (i.e., negative expert ratings and positive user ratings), we computed user-expert gap scores by subtracting the standardized user rating from the standardized expert rating. Such a method makes it possible to estimate the differences in standard deviation between the two systems, resulting in scores ranging from −5.42 to 6.11 standard deviations. Positive scores indicate that apps have higher expert ratings than user ratings relative to the other apps in the dataset, while negative scores indicate that apps have lower expert ratings than user ratings. However, this alone cannot help identify missed opportunities and overrated apps as the same user-expert gap score can result from different expert and user ratings. For example, two apps could have standardized expert ratings of 1 and −2, while having standardized user ratings of −1 and −4, respectively. This would lead to the same gap score of 2, although only the first health app has above-average expert rating. For this reason, missed opportunities were defined as apps with an above-average expert rating and a below-average user rating, as well as a user-expert gap of 1.5 or greater. Similarly, overrated apps were defined by a below-average expert rating and an above-average user rating, as well as a difference ≥1.5. The choice of 1.5 standard deviations was arbitrary.

## Results

### Differences Between Health Domains, Platforms and Number of Reviews

To provide a sensitive analysis of the impact of the number of reviews, we separated our sample into quartiles of about 300 health apps. We ran an ANOVA to examine existing differences in user and expert ratings between health domains (physical health, mental health), platforms (Android, iOS) and number of reviews (1st, 2nd, 3rd, 4th quartiles; ranges for each quartile are available in Table 2). User and expert ratings, user-expert gap scores, as well as data rights, usability, clinical and evidence subscales were entered as dependent variables (see Table 2). Since this study is likely overpowered due to the large sample size and should detect small effects, only moderate effects are reported (i.e., $\eta_{p}^{2}$ ≥ 0.06). There was no effect of health domain or platform on the different ratings, although there was a near-moderate effect of platform on user ratings, *F*_(1233, 1)_ = 55.44, *p* < 0.001, $\eta_{p}^{2}$ = 0.05. User ratings are higher on iOS (*M* = 4.36, *SD* = 0.76) than on Android (*M* = 4.09, *SD* = 0.60). There were moderate effects of the number of reviews on expert ratings, *F*_(1233, 3)_ = 25.04, *p* < 0.001, $\eta_{p}^{2}$ = 0.06, data rights subscale, *F*_(1233, 3)_ = 30.04, *p* < 0.001, $\eta_{p}^{2}$ = 0.07, usability subscale, *F*_(1233, 3)_ = 31.04, *p* < 0.001, $\eta_{p}^{2}$ = 0.07, and user ratings, *F*_(1233, 3)_ = 26.98, *p* < 0.001, $\eta_{p}^{2}$ = 0.06. For brevity, *post-hoc* analyses were performed with a Tukey test (α = 0.05) only for expert and user ratings. Expert ratings are higher in the 3rd and 4th quartiles than in the 1st and 2nd. Five-star user ratings are consistently higher in the 4th quartile than in the 1st, 2nd and 3rd quartiles. None of the interactions were significant.

**Table 2:** Comparison of user and expert ratings by health domain (physical health, mental health), platform (Android, iOS) and quartile in the number of reviews (1st, 2nd, 3rd, 4th).

	**Health domain**		**Platform**		**Number of reviews**			
	**Physical health**	**Mental health**	**Android**	**iOS**	**1st**	**2nd**	**3rd**	**4th**
User-expert gap	−0.01 (1.34)	0.02 (1.42)	0.18 (1.28)	−0.18 (1.45)	−0.25 (1.68)	0.13 (1.34)	0.18 (1.31)	−0.06 (1.02)
User rating	4.21 (0.71)	4.24 (0.69)	4.01 (0.60)	4.36 (0.76)	4.18 (0.89)	4.03 (0.70)	4.20 (0.64)	4.50 (0.39)
Expert rating	−0.09 (2.44)	0.10 (2.76)	−0.05 (2.59)	0.05 (2.58)	−0.82 (2.66)	−0.38 (2.49)	0.35 (2.48)	0.85 (2.40)
Data rights	3.08 (2.63)	3.08 (3.08)	3.08 (2.80)	3.08 (2.87)	2.07 (3.45)	2.85 (2.83)	3.40 (2.52)	3.98 (2.02)
Usability	3.62 (2.46)	3.55 (2.38)	3.48 (2.41)	3.70 (2.44)	2.90 (2.32)	3.02 (2.39)	3.93 (2.35)	4.52 (2.27)
Clinical	2.11 (2.39)	2.35 (2.60)	2.19 (2.49)	2.23 (2.49)	2.14 (2.47)	2.31 (2.42)	2.40 (2.65)	2.01 (2.40)
Evidence	0.30 (0.70)	0.39 (0.75)	0.35 (0.72)	0.34 (0.73)	0.23 (0.61)	0.27 (0.65)	0.36 (0.73)	0.51 (0.85)

*The expert rating is the sum of the standardized subscales. Each cell represents Mean (SD). 1st quartile: 1–21 reviews; 2nd: 22–174 reviews; 3rd: 175–3,691 reviews; 4th: 3,705–2,409,239 reviews*.

### Testing User-Expert Gaps

According to previous analyses, the following are carried out separately for each quartile and platform. Health domains are not included as they have not been shown to impact user and expert ratings. For health apps in the 1st quartile, Bayesian Pearson correlations between user and expert ratings yielded a Bayes factor of 6.14 on Android (*r* = −0.12) and 14.72 on iOS (*r* = −0.04). For the 2nd quartile, Bayes factor was 13.59 on Android (*r* = 0.05) and 12.21 on iOS (*r* = −0.06). For the 3rd quartile, Bayes factor was 15.29 on Android (*r* = 0.03) and 12.43 on iOS (*r* = 0.05). For the 4th quartile, Bayes factor was 3.52 on Android (*r* = 0.14) and 12.25 on iOS (*r* = 0.06). All Bayes factors reached the threshold indicating either moderate (value ≥ 3) or strong evidence (value ≥ 10) in favor of user-expert gaps (i.e., H0). For completeness, we performed a linear regression with user ratings as the dependent variable and all expert subscales entered simultaneously into the model as independent variables. We added the raw number of reviews as a covariate. Table 3 shows the details of the regression model. The model with all expert ratings subscales was a significant predictor of user ratings, although the effect size is small. Indeed, the model explained only 2.7% of the variance in user ratings, *F*_(5, 1227)_ = 6.84, *p* < 0.001. Data rights, usability and evidence were all significant predictors of user ratings, although again, the effect sizes are quite small. The number of reviews also predicted a significant, but small proportion of the variance.

**Table 3:** Regression analysis summary for expert rating subscales and number of reviews predicting user rating.

	***b***	***b* 95% CI (LL, UL)**	**β**	***t***	***p***	***r***	***p***
(Intercept)	4.11	(4.03, 4.18)		106.32	<0.001		
Data rights	0.02	(0.01, 0.03)	0.08	2.62	0.009	0.09	0.003
Usability	0.03	(0.01, 0.05)	0.10	3.40	<0.001	0.10	<0.001
Clinical	−0.01	(−0.03, 0.01)	−0.04	−1.34	0.18	−0.002	0.95
Evidence	−0.08	(−0.12, −0.01)	−0.07	−2.45	0.02	−0.06	0.06
Nb of reviews	0.00	(0.00, 0.00)	0.08	2.77	0.006	0.08	0.002

### Insights From a User-Expert Gap Metric

**Figure 1:**
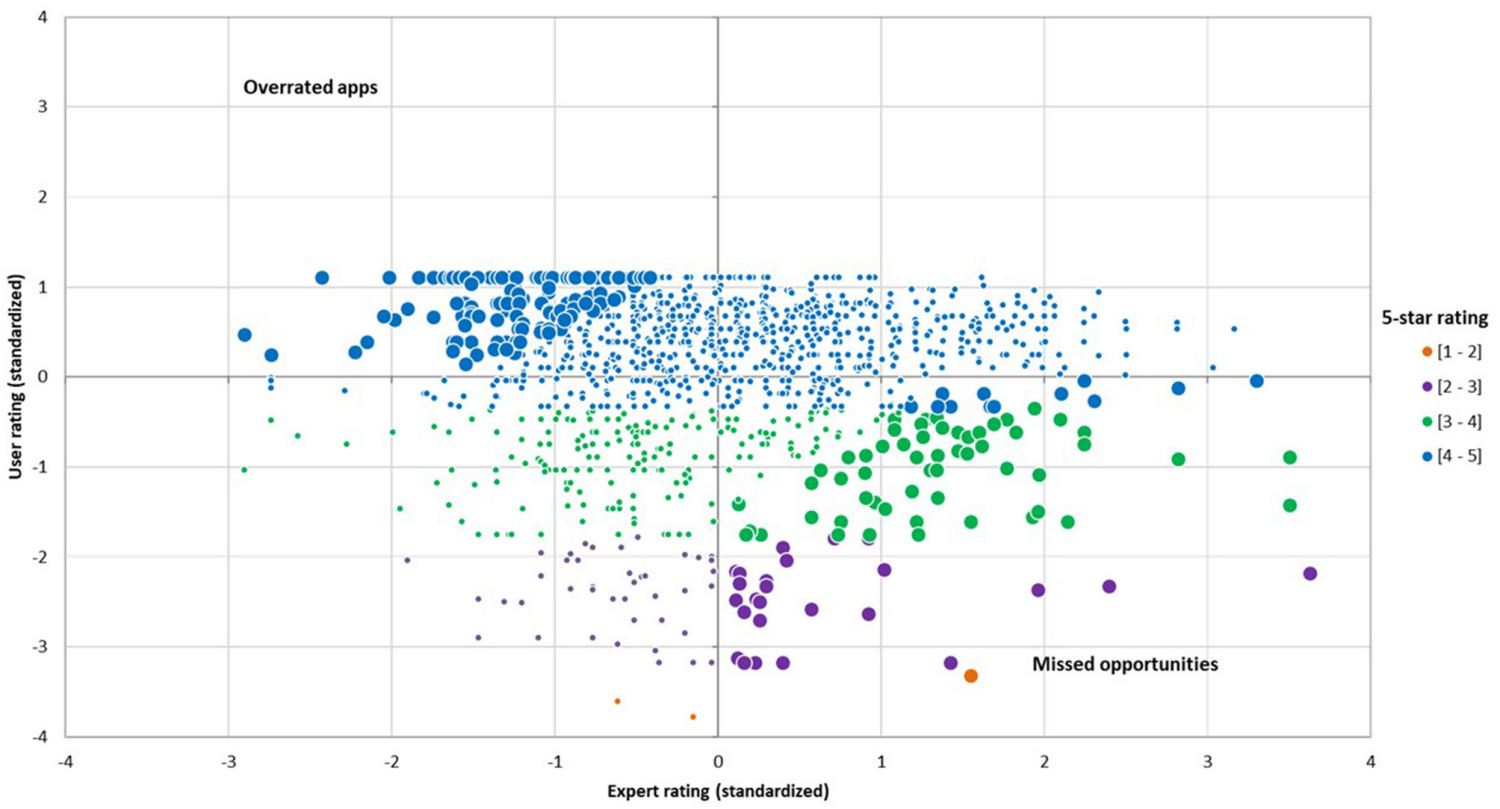
Distribution of standardized user and expert ratings. This figure shows user-expert gaps for each level of 5-star ratings and identifies missed opportunities and overrated apps.

As shown in Figure 1, a significant proportion of health apps were identified as missed opportunities (8.0%) or overrated (10.3%). It should be noted that a given health app may have a different user rating on iOS and Android, resulting in different user-expert gap scores. Therefore, an app can be identified as a missed opportunity (or as an overrated app) on one platform but not the other. Among the health apps that enjoy a high number of reviews, examples of overrated apps are *Blood Pressure Tracker*+ on iOS, a physical health app which has a five-star rating of 4.70 (4,200 reviews) and a user-expert gap of −2.73, and *Wim Hof Method* on Android, a physical health app which has a five-star rating of 4.71 (27,000 reviews) and a user-expert gap of −1.96. Examples of missed opportunities are *Fitbit* on Android, which has a five-star rating of 3.59 (800,000 reviews) and a user-expert gap of 3.73, and *Dexcom G6* on Android, a physical health app which has a five-star rating of 2.38 (6,000 reviews) and a user-expert gap of 3.56. Among the health apps with fewer reviews, examples of overrated apps are *Breathe Easy* on Android, a mental health app which has a five-star rating of 4.63 (70 reviews) and a user-expert gap of −2.13, and *Blood Pressure* ++ on iOS, a physical health app which has a five-star of 4.44 (80 reviews) and a user-expert gap of −1.68. Examples of missed opportunities are *Sleepio* on iOS, a mental health app which has a five-star rating of 2.70 (90 reviews) and a user-expert gap of 5.81, and *Aby* on Android, a physical health app which has a five-star rating of 3.60 (168 reviews) and a user-expert gap of 2.11.

## Discussion

### Evidence of User-Expert Gaps in Health App Ratings

This study aimed to examine the concordance between user ratings from commercial app stores and expert ratings found in Therappx's Core, a Canadian curated library of health apps designed for professionals. The way consumers and experts define quality is very often disconnected (9). In line with previous research (1, 2), this study revealed evidence in favor of a gap between the two rating systems. This is supported by the weak correlations between user ratings and expert rating subscales. For instance, the linear regression showed that a change of one unit in the data rights subscale is associated with only a small increase of about 0.02 units on the five-star user rating system. Although statistically significant, such a change in user ratings is unlikely to influence the choice of healthcare professionals. Evidence of user-expert gaps is strengthened by the fact that the number of reviews does not impact the relationship between five-star and expert ratings. This reinforces the need to provide consumers, healthcare professionals and other stakeholders with an alternative to star-rating that offers key privacy and clinical metrics. This study suggests that the information provided in curated libraries and commercial stores has little if any overlapping characteristics.

Curated libraries like Core and others have the potential to provide reliable information about clinical quality, utility, privacy, and security. These dimensions have been mentioned repeatedly as key indicators helping healthcare professionals to make a decision (9, 15). The five-star rating system for mobile applications is reputed to be flawed and this study again indicates that professionals need to be careful when incorporating this source of information into their decision-making process. A clear indication of reliability issues with user ratings is the overall difference seen between platforms. While our analyses did not allow pairwise comparisons between the iOS and Android versions of a health app, it does suggest that iOS and Android users rate apps differently, which is not an issue with expert ratings. These differences between platforms are in line with past research (11). Another problem with user reviews is that they are biased in favor of higher five-star values. Therefore, such a metric offers little value in truly distinguishing between apps.

### Actionable Insights From a New User-Expert Gap Metric

This study contributes to the health app literature and care delivery by providing a new user-expert gap metric, which in turn can be used to capture unique opportunities resulting from the different assessments made by users and experts. By standardizing both user and expert ratings, this helps to compare health apps on a similar scale relative to other apps. This also helps nuance the strength of the user-expert gap, as the distribution of scores shows that roughly 80% of health apps actually have similar user and expert ratings, as defined by a difference of <1.5 standard deviations. Strong user-expert rating gaps were observed with a subset of apps. More importantly, the practical importance of such a metric is the potential to identify missed opportunities in commercial stores, as well as apps favorably rated by users, but poorly rated by experts, i.e., overrated apps. These health apps represented about 20% of the sample.

This has important implications for research and practice. For example, there are several studies examining the correlates of highly rated health apps, with some researchers focusing exclusively on 4- to 5-star apps (16). As shown by the user-expert gap scores, this can lead to missed opportunities, both in research and in the clinical setting. Professionals and consumers who still favor searches in commercial app stores to find health apps are likely to miss high-quality apps as assessed by experts. In fact, several missed opportunities have between 25 and 100 reviews, which does not allow for a high-level rank in the app proposal algorithm of commercial app stores. These health apps are often developed by non-profits that may not have the marketing power to improve exposure. This is important because most of the missed opportunities identified by the user-expert gap metric are supported by clinical or feasibility studies, a key criterion mentioned by healthcare professionals. Only about 20% of the health apps in the sample have been submitted to a feasibility study or clinical trial investigating their use. Such information is not readily available to consumers, as developers who have conducted studies do not always share this fact in descriptions provided in commercial app stores (4). By focusing on five-star ratings and number of downloads, commercial stores might suffer from discoverability issues. As the short list of health apps we have provided shows, the user-expert gap scores are higher for missed opportunities than for overrated apps, suggesting that Core and other curated libraries might be especially useful for discovering overlooked apps with fewer reviews and exposure.

Similarly, consumers and professionals searching commercial app stores for discovering health apps should further their investigation into the overrated apps identified by a user-expert gap metric before using or prescribing it. Despite having high five-star ratings from users, these apps may have limited design quality, problematic data or privacy management, and may lack key clinical features. Curated libraries can add value by helping healthcare professionals find information to help them decide among several health apps. However, a discrepancy between user and expert ratings should not be interpreted as a discrepancy between expert rating and value of an app to the consumer. The ability of Core and other curated libraries to produce meaningful, clinically relevant ratings that lead to uptake and use of mobile health apps should be assessed in future efficacy studies.

### Rethinking the Delivery of Digital Health Care

Use of health apps in general practice and health services is starting to receive increasing interest from consumers, but concrete actions from healthcare professionals and stakeholders are often lagging behind. There are few evidence-based guidelines to assist adoption and prescription of health apps by professionals, or to help integrate mobile interventions into health services. However, initiatives are on the rise. For example, Melcher and Torous identified twenty-six college counseling centers offering health apps to students on their website (17). Unfortunately, they concluded that most of the recommended apps were neither safe, supported by evidence nor up-to-date. One of the most common barriers to adoption is the difficulty of keeping up with the rapid pace of the industry, which could in fact be overcome by providing access to curated libraries.

Under the right circumstances, incorporating into the general practice a curated health apps library is feasible and might help drive uptake. An opinion study conducted with 600 Spanish nurses revealed that 97% believe that health apps should be certified and that 50% would be willing to prescribe them if approved by their institution (18). Providing access to a curated library could help meet the need for professionals to obtain a seal of approval before prescribing an app, while also making it easier to discover some high-quality health apps that would otherwise be overlooked by searching in commercial app stores. In line with this, a recent study showed positive results on prescribing habits by providing Australian GPs with a library of six health apps, including Smiling Mind, an app for mental health (19). Smiling Mind's five-star rating is 3.80 on Android and 4.50 on iOS, which in the current study led to a user-expert gap score of 2.43 and 1.43, respectively. Core identifies Smiling Mind as a missed opportunity on Android. Likewise, stepped-care models in mental health, which are used in the UK (20) and Canada (21), clearly articulate which health apps should be considered, as well as why and when they should be recommended or used by healthcare professionals. For example, the Government of Newfoundland and Labrador (Canada) has integrated TAO mobile as part of its stepped-care model. Again, this app is one of the top missed opportunities in the Core database. With only <50 reviews, TAO Mobile's five-star rating is 3.10 on Android and 1.90 on iOS, which led to a user-expert gap score of 3.16 and 4.87, respectively. Thus, implementing stepped-care models with a digital approach and other digital health programs on the institutional level could help professionals prescribe high-quality apps that are often overlooked (22).

### Strengths and Limitations

One important strength of this study is its sample size, which is significantly higher than similar studies (1, 12, 23), although it should be noted that this includes overlaps of the same health apps between stores. This, combined with the fact that both physical health and mental health are covered, makes this study one of the most comprehensive estimates of the relationship between user and expert ratings across the marketplace. The user-expert gap metric exposed new insights into finding missed opportunities in commercial app stores, while also revealing several overrated health apps.

However, key limitations include the lack of consideration for accessibility and cost, which are important parameters that must be considered when discussing the use of health apps by consumers. In addition, it should be noted that the Core database was developed for the North American market, for English and French speaking consumers and professionals. All the health apps featured in this study are available for download in the United States and Canada, although many apps are developed in another country. This certainly impacts the external validity of the study, although there is no reason to believe that the user-expert gaps would be different from one country to another. Finally, this study relied on a specific curated library (Core), but there are many more as mentioned earlier. The lack of reliability between curated libraries has already been criticized (24), so it would be important to examine the reliability of user-expert gaps across multiple curated libraries. Finally, user ratings tend to change overtime, and experts are assessing apps in Core at every major update. Current results, such as health apps identified as overrated or missed opportunities, may differ in the future.

## Conclusion

We have provided clear evidence of a mismatch between five-star user ratings and expert ratings as found in Core. There is growing interest from stakeholders in using curated libraries to help integrate digital health into clinical practice and health institutions. This study shows that user-expert gaps derived from these libraries could empower stakeholders with the ability to select generally overlooked health apps and avoid overrated ones.

## Data Availability Statement

The raw data supporting the conclusions of this article will be made available by the authors, without undue reservation.

## Author Contributions

P-LdC designed the study, performed statistical analysis, and drafted the manuscript. AC and MC assisted in interpretation of the data. AC, MC, and JF contributed to critically revising the manuscript. AG organized the dataset. All authors contributed to manuscript revision, read, and approved the submitted version and accept accountability for all aspects of the accuracy and integrity of the work.

## Funding

This study received funding from Therappx as part of a pilot program funded by Quebec's Ministry of Economics and Innovation.

## Conflict of Interest

All of the authors are or have been involved in the business of Therappx. P-LdC is currently Assistant Professor at Université du Québec à Montréal, but was employed by Therappx as Head of Research. P-LdC is now a clinical advisor for the company. AC is currently CEO. MC is currently CCO. JF is currently clinical advisor and content moderator. AG is currently CTO.

## Publisher's Note

All claims expressed in this article are solely those of the authors and do not necessarily represent those of their affiliated organizations, or those of the publisher, the editors and the reviewers. Any product that may be evaluated in this article, or claim that may be made by its manufacturer, is not guaranteed or endorsed by the publisher.

## Footnotes

^1^Available online at https://psychiatry.org/psychiatrists/practice/mental-health-apps (accessed July 26, 2021).

^2^Available online at https://appfinder.orcha.co.uk/ (accessed July 26, 2021).

^3^Available online at https://onemindpsyberguide.org/ (accessed July 26, 2021).

^4^Available online at https://mindtools.io/ (accessed July 26, 2021).

^5^Available online at https://app.therappx.com/ (accessed July 26, 2021).

^6^Available online at https://blog.Therappx.com/the-reliability-of-Therappx-core-today-and-tomorrow/ (accessed July 26, 2021).
